# PBTK Modeling Demonstrates Contribution of Dermal and Inhalation Exposure Components to End-Exhaled Breath Concentrations of Naphthalene

**DOI:** 10.1289/ehp.9778

**Published:** 2007-02-14

**Authors:** David Kim, Melvin E. Andersen, Yi-Chun E. Chao, Peter P. Egeghy, Stephen M. Rappaport, Leena A. Nylander-French

**Affiliations:** 1 Department of Environmental Sciences and Engineering, School of Public Health, The University of North Carolina at Chapel Hill, Chapel Hill, North Carolina, USA; 2 CIIT Centers for Health Research, Research Triangle Park, North Carolina, USA

**Keywords:** dermal, exposure assessment, inhalation, jet fuel, naphthalene, physiologically based toxicokinetic model

## Abstract

**Background:**

Dermal and inhalation exposure to jet propulsion fuel 8 (JP-8) have been measured in a few occupational exposure studies. However, a quantitative understanding of the relationship between external exposures and end-exhaled air concentrations has not been described for occupational and environmental exposure scenarios.

**Objective:**

Our goal was to construct a physiologically based toxicokinetic (PBTK) model that quantitatively describes the relative contribution of dermal and inhalation exposures to the end-exhaled air concentrations of naphthalene among U.S. Air Force personnel.

**Methods:**

The PBTK model comprised five compartments representing the stratum corneum, viable epidermis, blood, fat, and other tissues. The parameters were optimized using exclusively human exposure and biological monitoring data.

**Results:**

The optimized values of parameters for naphthalene were *a*) permeability coefficient for the stratum corneum 6.8 × 10^−5^ cm/hr, *b*) permeability coefficient for the viable epidermis 3.0 × 10^−3^ cm/hr, *c*) fat:blood partition coefficient 25.6, and *d*) other tissue:blood partition coefficient 5.2. The skin permeability coefficient was comparable to the values estimated from *in vitro* studies. Based on simulations of workers’ exposures to JP-8 during aircraft fuel-cell maintenance operations, the median relative contribution of dermal exposure to the end-exhaled breath concentration of naphthalene was 4% (10th percentile 1% and 90th percentile 11%).

**Conclusions:**

PBTK modeling allowed contributions of the end-exhaled air concentration of naphthalene to be partitioned between dermal and inhalation routes of exposure. Further study of inter- and intraindividual variations in exposure assessment is required to better characterize the toxicokinetic behavior of JP-8 components after occupational and/or environmental exposures.

The single largest source of chemical exposure on military bases of the North Atlantic Treaty Organization (NATO) is jet propulsion fuel 8 (JP-8), which is the preferred fuel for both aircraft and military vehicles in NATO countries. JP-8 comprises many aromatic hydrocarbons, including benzene and naphthalene, and aliphatic hydrocarbons such as nonane and decane (McDougal et al. 2000). Exposures to JP-8 can occur during spills, transportation and storage of the fuel, as well as during fueling, general maintenance and operation of aircraft and military vehicles, fueling of military tent heaters, and cleaning and degreasing of parts with the fuel.

Since JP-8 can enter the body via both inhalation and dermal contact, the assessment of occupational exposures to fuel constituents can be difficult. Personal sampling of JP-8 vapors provides information about inhalable levels but not about dermal exposure levels. Similarly, sampling the exposed skin provides information about dermal but not about inhalable levels. Conversely, the collection of end-exhaled breath concentrations provides an integrated estimate of uptake via both inhalation and dermal contact (Egeghy et al. 2003; Pleil et al. 2000) but cannot determine the relative contributions of the two exposure routes to the internal dose. Through statistical evaluation of levels of naphthalene in air, breath, and skin, measured in the U.S. Air Force personnel during fuel maintenance procedures, both inhalation and dermal exposures to JP-8 were demonstrated to contribute to the internal dose (Chao et al. 2006). However, because of the respiratory protection used in that population, it was difficult to determine the relative contributions of dermal and inhalation exposures to the systemic levels of JP-8 components.

Physiologically based toxicokinetic (PBTK) modeling is an effective tool for quantifying the absorption, distribution, metabolism, and elimination of chemicals. PBTK models have been developed for various components of JP-8, notably naphthalene and decane (Perleberg et al. 2004; Quick and Shuler 1999; Willems et al. 2001). The model developed by Quick and Shuler (1999) focused on the disposition of naphthalene in five compartments representing the lungs, liver, fat, rapidly perfused tissues, and slowly perfused tissues and relied on *in vitro* data to calibrate kinetic constants. Willems et al. (2001) refined the Quick and Shuler (1999) model by using kinetic constants derived from *in vivo* data from laboratory animal experiments performed by the National Toxicology Program. They observed that a diffusion-limited PBTK model was necessary to characterize the toxicokinetic behavior of naphthalene in rats and mice. Perleberg et al. (2004) developed a PBTK model using decane as a chemical marker of JP-8. Data for calibration and validation of this model were derived from an animal study in which rats were exposed for 4 hr to decane vapor at three different concentrations (1,200, 781, or 273 ppm). Their final model consisted of flow-limited compartments for liver and lung, and diffusion-limited compartments for brain, bone marrow, fat, skin, and spleen. The model predicted the time course of decane in tissue and blood from low-level exposures to decane vapor.

Because the PBTK models mentioned above did not examine the uptake via skin, we developed a PBTK model that included both inhalation and dermal routes of exposure. Naphthalene was chosen as the surrogate for JP-8 exposure because it is abundant in JP-8, is readily absorbed into blood, and is only a minor component in confounding sources of exposure such as cigarette smoke and gasoline exhaust (Rustemeier et al. 2002; Serdar et al. 2003). We expanded on the structure of a data-based compartmental model that was used to quantify the absorption, distribution, and elimination of jet fuel components (Kim et al. 2006b). Data from a study of controlled dermal exposure in humans were used to optimize the parameters in the PBTK model (Kim et al. 2006a). The optimal PBTK model, combined with exposure and biomarker data from field studies (Chao et al. 2005; Egeghy et al. 2003), was used to quantify the relative contributions of dermal and inhalation exposures to end-exhaled breath concentrations of naphthalene among U.S. Air Force personnel.

## Materials and Methods

### Laboratory study of dermal exposure to JP-8

We conducted a laboratory study to quantify the dermal absorption and penetration of JP-8 components across human skin *in vivo* (Kim et al. 2006a). Approval for this study was obtained from the Office of Human Research Ethics (School of Public Health, The University of North Carolina at Chapel Hill, Chapel Hill, North Carolina). Written informed consents were received from all study volunteers. The study consisted of 10 volunteers (5 females and 5 males) recruited for this study. Exposures were conducted in an exposure chamber. One forearm was placed palm up inside the exposure chamber, and two aluminum application wells were pressed against the skin and sealed for the duration of the experiment (0.5 hr). At the end of the 0.5-hr exposure period, the exposed sites were tape-stripped 10 times with adhesive tape strips. Tape strips were used to quantify the mass of naphthalene in successive layers of the stratum corneum. Both tape-strip and blood samples were analyzed by gas chromatography–mass spectrometry (GC-MS). The time course of naphthalene in blood for all study volunteers showed considerable interindividual variability. For example, the time course for a 23-year-old Caucasian male with a body mass index (BMI) of 25 kg/m^2^ was very different from that of a 24-year-old Caucasian female with a BMI of 22 kg/m^2^. For the male volunteer, the maximum concentrations in blood (*C*_max_) occurred shortly after the end of exposure (*t*_max_ ≈ 30 min), with a value of 0.8 ng/mL. The *C*_max_ for the female volunteer occurred at *t*_max_ ≈ 60 min, with a value of 0.3 ng/mL. In either case, the concentrations in blood at *t* > 0 min did not return to baseline levels.

### Field study of dermal and inhalation exposures to JP-8

Exposure data were obtained from the assessment of dermal and inhalation exposures to JP-8 in the personnel at six U.S. Air Force bases in the continental United States (Chao et al. 2005; Egeghy et al. 2003). The duration of exposure was approximately 4 hr. The concentration of naphthalene in the personal breathing-zone air (referred to as “air concentration” in this article) was measured using passive monitors (Egeghy et al. 2003). End-exhaled breath samples were collected pre-and postexposure (Egeghy et al. 2003). The end-exhaled breath measurements are indicative of alveolar air (Egeghy et al. 2000). Both air and breath samples were analyzed by GC with photoionization detection. Tape strips were used to quantify dermal exposure to naphthalene at specific body regions; results were extrapolated to the total surface area of skin to estimate whole-body dermal exposure to naphthalene. We collected dermal samples postexposure using adhesive tape strips with the dimension of 2.5 cm × 4.0 cm (surface area 10 cm^2^) from exposed body regions including the forehead, neck, shoulders, arms, hands, legs, knees, feet, and buttocks (Chao et al. 2005). Tape-strip samples were extracted with acetone and analyzed by GC-MS.

The median air concentrations of naphthalene in air samples were 1.9 μg/m^3^ (range, < 1.0–16.9 μg/m^3^), 29.8 μg/m^3^ (range, < 1.0–932 μg/m^3^), and 867 μg/m^3^ (range, 12.8–3,910 μg/m^3^) for the low-, medium-, and high-exposure groups, respectively (Egeghy et al. 2003). The median preexposure breath levels of naphthalene were < 0.5 μg/m^3^ (range, < 0.5–36.3 μg/m^3^), < 0.5 μg/m^3^ (range, < 0.5–16.1 μg/m^3^), and < 0.5 μg/m^3^ (range, < 0.5–6.1 μg/m^3^) for the low-, medium-, and high-exposure groups, respectively. The median postexposure breath levels were 0.73 μg/m^3^ (range, < 0.5–6.9 μg/m^3^), 0.93 μg/m^3^ (range, < 0.5–13.0 μg/m^3^), and 1.83 μg/m^3^ (range, < 0.5–15.8 μg/m^3^) for the low-, medium-, and high-exposure groups, respectively. The corresponding median concentrations of dermal samples were 344 ng/m^2^ (range, 159–54,200 ng/m^2^), 483 ng/m^2^ (range, 150–13,200 ng/m^2^), and 4188 ng/m^2^ (range, 100–4,880,000 ng/m^2^) in the low-, medium-, and high-exposure groups, respectively (Chao et al. 2005).

### Description of the PBTK model

**Figure 1:**
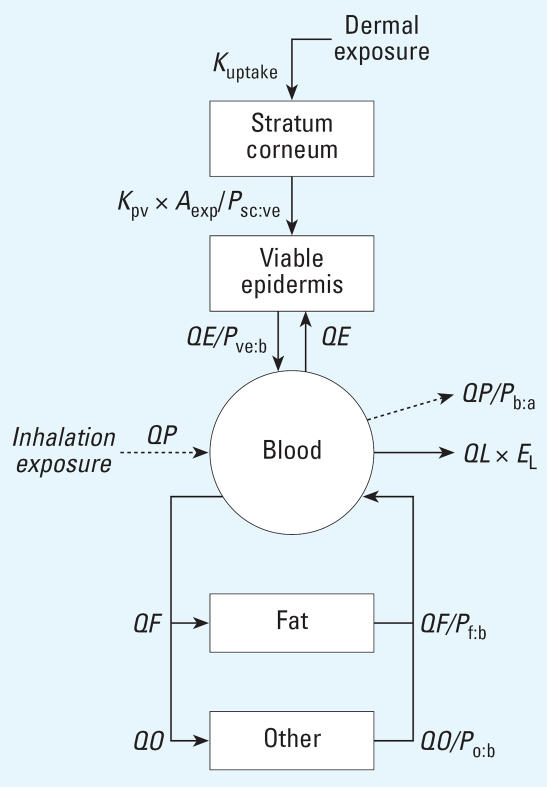
Schematic of the physiologically based toxicokinetic (PBTK) models for the study of naphthalene toxicokinetics. Pulmonary uptake of naphthalene in the personal breathing-zone and pulmonary clearance from the blood compartment are added to a previously published dermatotoxicokinetic model (Kim et al. 2006b). Abbreviations in the PBTK model: *K*_uptake_, input rate constant for dermal exposure; *K*_pv_, permeability coefficient for the viable epidermis; *A*_exp_, exposed surface area; *P*_sc:ve_, stratum corneum:viable epidermis partition coefficient; *QE*, blood flow rate to skin; *P*_ve:b_, viable epidermis:blood partition coefficient; *QP*, pulmonary ventilation rate; *P*_b:a_, blood:air partition coefficient; *QF*, blood flow rate to fat; *P*_f:b_, fat:blood partition coefficient; *QO*, blood flow rate to other tissue; *P*_o:b_, other tissue:blood partition coefficient; *E*_L_, extraction ratio.

A dermatotoxicokinetic (DTK) model, which was previously developed for describing the disposition of aromatic and aliphatic components of JP-8 after controlled dermal exposure (Kim et al. 2006b), formed the basis of the PBTK model (Figure 1). The DTK model consisted of five compartments representing the surface, stratum corneum, viable epidermis, blood, and storage tissues. The parameters for the DTK model were estimated by fitting the model to the data. The major difference between the DTK and the PBTK model structures is that the storage compartment was split into fat and all other tissues. The rationale for defining the storage compartment in this fashion was based on the high fat:blood partition coefficient (*P*_f:b_) of naphthalene (160), which is more than 5 times the partition coefficient of the other tissues (Fiserova-Bergerova 1983). Further additions to the PBTK model included pulmonary uptake and clearance. The skin compartments were composed of the skin directly under the exposed area. All tissues were perfusion limited and well mixed. Absorbed naphthalene was distributed to other tissue compartments at a rate equal to the rate of blood flow to that tissue. Naphthalene was stored in the fat and other tissue compartments based on the physiologic parameters of that compartment (i.e., tissue:blood partition coefficient, tissue volume, and blood perfusion rate).

Most physiologically based compartmental models separate the arterial blood from the central venous blood, whereas data-based compartmental models treat the blood as one compartment. Also in data-based compartmental models, the peripheral compartments represent organs or tissues that, being poorly perfused with blood, are in slower equilibrium distribution with blood. Blood samples were collected from the antecubital vein in the study by Kim et al. (2006a). The antecubital vein drains blood from the hand and the superficial layers of the forearm. The concentration of solute in the antecubital vein is different from the concentrations of solute in the arterial and central venous blood (Levitt 2004). However, the blood in the antecubital vein is in rapid equilibrium with arterial and central venous blood relative to the fat and other tissue compartments. Therefore, we treated the arterial and central vein as a single compartment, and approximated the concentration of naphthalene in the central (i.e., blood) compartment using measurements made from the antecubital vein.

Two routes of exposure were modeled: dermal and inhalation. Pulmonary uptake is equal to the pulmonary ventilation rate (QP) times the concentration of naphthalene in the personal breathing-zone (*C*_PBZ_):


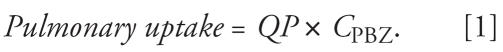


In Equation 1, rapid equilibration of naphthalene occurs across the alveolar lining, and neither storage nor metabolism in the lungs appreciably affects the uptake of naphthalene into the systemic circulation. Because arterial, lung, and venous blood are treated as a combined blood compartment, the rate of absorption is equal to pulmonary uptake. Dermal absorption and penetration is modeled as a one-directional diffusive process according to Fick’s first law of diffusion. As such, the diffusion of naphthalene across the stratum corneum (SC) and the viable epidermis (VE) are quantified using permeability coefficients, the area of exposure, and the thickness of the membrane (McCarley and Bunge 2001; McDougal and Boeniger 2002). The rate of efflux from the SC to the VE is dependent on the solubility of naphthalene in the SC relative to the VE. Therefore, the rate of efflux of naphthalene from the SC to the VE is equal to *K*_pv_ × *A*_exp_ × *CD/P*_sc:ve_, where *K*_pv_ is the permeability coefficient across the VE, *CD* is the concentration in the SC, *A*_exp_ is the exposed surface area, and *P*_sc:ve_ is the SC:VE partition coefficient. The mass balance differential equation (MBDE) for the SC is


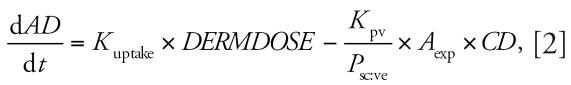


where *K*_uptake_ is the input rate constant and *DERMDOSE* is the dose to the skin. The rate of input from blood to VE is the cutaneous blood flow rate (QE) times the concentration of naphthalene in blood (CB), and the rate of efflux from the VE to blood is controlled by QE and the solubility of naphthalene in the blood (*P*_ve:b_). The MBDE for the amount of naphthalene in the VE is


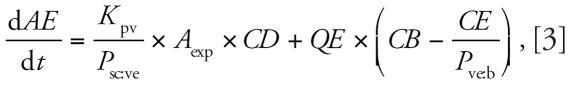


where *AE* is the amount and *CE* is the concentration of naphthalene in the VE.

Elimination of naphthalene proceeds by two significant mechanisms: exhalation and metabolism. The concentration of naphthalene in end-exhaled air is equal to the blood concentration divided by the blood:air partition coefficient (*P*_b:a_). Pulmonary clearance of naphthalene is *QP* divided by *P*_b:a_. Metabolism of naphthalene occurs in the liver by a single metabolic pathway following first-order kinetics. The initial step in naphthalene metabolism is the formation of naphthalene-1,2-oxide by cytochrome P450 monooxygenases [Agency for Toxic Substances and Disease Registry (ATSDR) 1995]. Liver clearance (*Cl_L_*) is


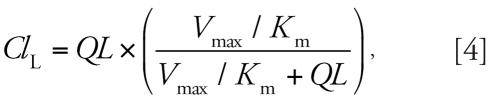


where *V*_max_ (millligrams per minute) is the maximum rate of metabolism, *K_m_* (milligrams per liter) is the Michaelis-Menten constant, and QL the blood flow rate to the liver (liters per minute). The ratio of liver clearance to liver blood flow is the extraction ratio (*E*_L_) where *E*_L_ is


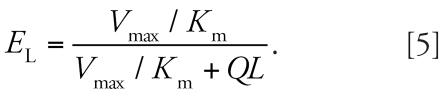


### Determination of the blood:air partition coefficient

Initial sensitivity analysis revealed that the concentration of naphthalene in end-exhaled air was highly sensitive to *P*_b:a_. Therefore, we measured *P*_b:a_ by equilibrating human blood with a known concentration of naphthalene (Gargas et al. 1989). Samples were analyzed with a Combi Pal autosampler configured for headspace analysis (CTC Analytics, Zwingen, Switzerland). A series of 20-mL crimp seal vials (MicroLiter Analysis Supplies, Suwanee, GA, USA) containing blood (test), air (reference), and a known amount of naphthalene (gas) were used in the experiment. The test and reference vials underwent the same process. First, the vial was heated to a temperature of 37°C, and a vent tool (LEAP Technologies, Carrboro, NC, USA) was used to equilibrate the pressure between the test/reference vials and the room. Next, a 2.5-mL gas-tight syringe was used to draw from the vial 1 mL of air, which was injected into the room air. Then, 1 mL of gas from the gas vial was transferred to the test/reference vial. The test/reference vial was kept at 37°C and agitated for 1 hr; we determined, by adjusting the incubation period, that 1 hr was the optimal time for achieving equilibrium. After incubation, a 1-mL sample was extracted from the test/reference vial and injected into the GC-MS for analysis. All analyses were conducted in triplicate. A six-point standard curve (*R*^2^ = 0.999) was used for quantitation of the naphthalene concentration in the test and reference vials. Partition coefficients were determined using Equation 6 (Gargas et al. 1989).


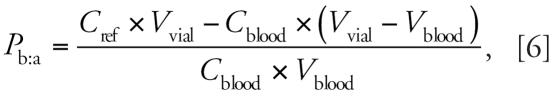


where *C*_ref_ is the naphthalene concentration in the reference vial, *V*_vial_ is the volume of the reference vial (20 mL), *C*_blood_ is the naphthalene concentration in the headspace of the test vial, and *V*_blood_ is the volume of blood (2 mL). Using Equation 6, we calculated a *P*_b:a_ value of 10.3.

### Model optimization

All physiologic parameters (cardiac output, ventilation rate, blood flow rate to the tissues, and tissue volumes) for humans were obtained from the literature (Brown et al. 1997). Other tissue partition coefficients were predicted from the octanol–water partition coefficients and regression models for different tissues (Abraham et al. 1985; Fiserova-Bergerova et al. 1984; Hansch et al. 1995; Willems et al. 2001). The maximum rates of naphthalene metabolism (*V*_max_) and Michaelis-Menten constant (*K_m_*) have been estimated for rats and mice (Willems et al. 2001). In our study, the rate of metabolism was assumed to follow first-order kinetics, given the relatively low naphthalene concentrations measured in postexposure breath samples (Egeghy et al. 2003). Initial sensitivity analysis revealed that the concentration of naphthalene in end-exhaled breath was not sensitive to *V*_max_*/K_m_*. Therefore, the parameters *K*_uptake_ , *K*_pv_ , *P*_f : b_ , and *P*_o : b_ (other tissue:blood partition coefficient) were adjusted to fit the blood time course data for each volunteer in the laboratory study. Initial values of all parameters were obtained from the literature (Guy and Potts 1992; McCarley and Bunge 2001; Qiao et al. 2000; Willems et al. 2001; Williams and Riviere 1995). The Nelder-Mead algorithm, with tolerance set at 1 × 10^−5,^ was used to optimize the parameters (Xcellon 2004).

### Comparison of dermal and inhalation routes of exposure

The Air Force data set was used to compare the relative contribution of dermal exposure with the end-exhaled breath concentration of naphthalene. The data set included personnel from the U.S. Air Force who had both dermal and inhalation exposures to JP-8 (Chao et al. 2005; Egeghy et al. 2003). From the Air Force personnel, end-exhaled breath samples were collected immediately at the end of the work shift and, later, at a central testing site (CTS). Three Air Force personnel were selected who represented the 10th, 50th, and 90th percentiles based on their end-exhaled breath measurements. The group had regular contact with jet fuel, and consisted of fuel-cell maintenance workers who entered fuel tanks during their work. Thus, the concentration of naphthalene in the air was much higher in the immediate work environment for these individuals compared with personnel who had no direct contact with JP-8 and, therefore, represented background exposures to naphthalene. The air concentration of naphthalene was reported for the duration of the sampling period, which included travel time to the CTS (~ 30 min). Thus, the air concentration at the work site was estimated as


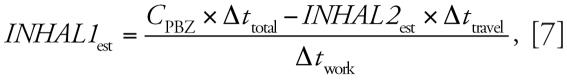


where *INHAL1*_est_ is the estimated concentration of naphthalene in the breathing zone during the work shift of Δ*t*_work_ hr, *C*_PBZ_ is the air concentration measured during the full sampling period of Δ*t*_total_ hr, *INHAL2*_est_ is the estimated background air concentration of naphthalene (i.e., air measurements from the U.S. Air Force personnel who had no direct contact with JP-8), and Δ*t*_travel_ is the time required to travel from the workplace to the CTS. The concentrations of naphthalene in the air and dermal samples and the duration of exposure were used as input terms for the PBTK model. Predicted end-exhaled breath concentrations were compared with measured levels of naphthalene in end-exhaled breath.

### Sensitivity analysis

Sensitivity analyses were performed to evaluate the relative importance of model parameters on the concentration of naphthalene in end-exhaled breath. Normalized sensitivity coefficients (NSC) were calculated using Equation 8 (Evans and Andersen 2000):


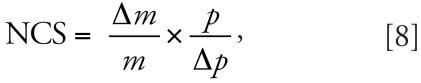


where *m* is the response variable (i.e., concentration of naphthalene in end-exhaled breath), Δ*m* is the change in the response variable, *p* is the value of the parameter of interest (e.g., blood:air partition coefficient), and Δ*p* is the change in the parameter value. Each parameter was changed 1% (i.e., Δ*p* ÷ *p* = 0.01).

## Results

### Dermal exposure toxicokinetics

**Figure 2:**
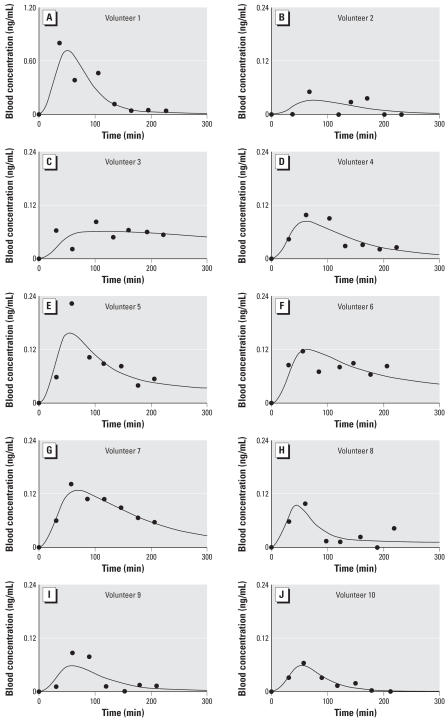
Plots comparing the PBTK model simulations to experimentally measured naphthalene concentrations in blood from 10 study volunteers (*A–J*) who were dermally exposed to JP-8 on the volar forearm.

The PBTK model was optimized for dermal exposure using data from 10 individuals who were exposed to JP-8 on the skin under laboratory conditions. The average height and weight of the subjects to whom JP-8 was administered on the skin was 174 cm and 61 kg, respectively (BMI = 21 kg/m^2^). Time-course plots showed considerable variability among the study volunteers (Figure 2). The mean ± SD of the peak concentration of naphthalene in blood was 0.18 ± 0.22 ng/mL and occurred at 62 ± 16 min. The time course for subject no. 1 was very different from that of the other volunteers. The peak concentration for this volunteer was 0.80 ng/mL and occurred at 37 min. Model predictions of the blood concentration of naphthalene are also shown for each volunteer using optimized parameter values in Figure 2. The skin parameters (*K*_uptake_ and *K*_pv_) and the partition coefficients *P*_f:b_ and *P*_o:b_ were adjusted to fit the blood time-course data for dermal exposures only; the optimal values are reported in Table 1. The rate of input from dermal exposure is equivalent to the product of the permeability coefficient for the SC (*K*_ps_), the exposed surface area (*A*_exp_), and the concentration of the naphthalene in JP-8 (*C*_JP-8_) (McCarley and Bunge 2001; McDougal and Boeniger 2002):


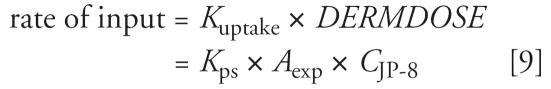


Equation 9 can be rearranged to solve for *K*_ps_ as follows:


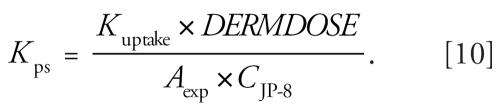


The optimized value of *K*_uptake_ is 0.031 ± 0.056 hr^−1^ (mean ± SD), and for *K*_ps_ it is 6.8 × 10^−5^ ± 5.8 × 10^−5^ cm/hr (mean ± SD) (Table 2). The sensitive parameters in the dermal only model were *DERMDOSE* (NSC = 1.0), *A*_exp_ (NSC = 1.0), *K*_uptake_ (NSC = 1.0), and P_o:b_ (NSC = −0.3).

### Prediction of end-exhaled breath concentrations

The optimized PBTK model was used to predict the end-exhaled breath concentration of naphthalene for 53 U.S. Air Force personnel (13 females and 40 males) who did not have dermal contact with jet fuel and had naphthalene end-exhaled breath concentrations > 0.0 μg/m^3^. The median height and weight of the personnel were 175 cm (range, 155–193 cm) and 77 kg (range, 52–116 kg), respectively. In the simulation, the median air concentration of naphthalene was 2.4 μg/m^3^ (range, 0.7–481.7 μg/m^3^) and was held constant for the duration of exposure (median duration, 237 min). For each U.S. Air Force subject, the preexposure concentration of naphthalene in the end-exhaled breath was subtracted from the postexposure measurements. The predicted concentration of naphthalene in end-exhaled breath (0.5 μg/m^3^) was the same as the median of the measured values. In addition, comparisons were made between measured and predicted concentrations of naphthalene in end-exhaled breath for each U.S. Air Force subject, using information on the subject’s height, weight, air concentration of naphthalene, and duration of exposure. The median relative difference between measured and predicted values was 26% (10th–90th percentile range, −71 to 196%).

**Figure 3:**
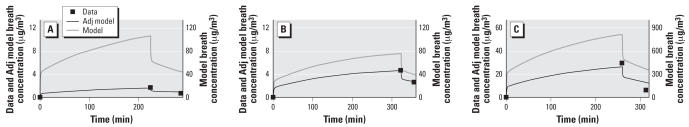
Model simulations and end-exhaled breath concentrations for the U.S. Air Force personnel who were exposed to JP-8 via inhalation and dermal routes. Breath samples were collected immediately at the end of the work shift and at a central testing site. Shown are the measured and predicted values for three U.S. Air Force personnel who represented the 10th (*A*), 50th (*B*), and 90th (*C*) percentiles of measured end-exhaled breath concentrations. Simulations are also shown after adjusting the air concentration of naphthalene during work to better estimate the true inhalation exposure [adjusted (Adj) model].

Model predictions of the end-exhaled breath concentration of naphthalene were compared with field measurements among three Air Force personnel who represented the 10th, 50th, and 90th percentiles based on their end-exhaled breath measurements. These three U.S. Air Force personnel spent time in a fuel tank during their work shift and were exposed to JP-8 on the skin. The input parameters and values for each U.S. Air Force personnel are reported in Table 3. The PBTK model consistently overpredicted the end-exhaled breath concentrations at the end of work shift for all three U.S. Air Force personnel (Figure 3). This could be attributed to the use of supply-air respirators. Therefore, the air concentration of naphthalene during work (i.e., *INHAL1*_est_) was adjusted (i.e., *INHAL1*_adj_) to estimate the likely inhalation exposure (Figure 3). The values of *INHAL1*_adj_ are reported in Table 4.

### Comparison of dermal and inhalation exposure routes

Simulations were conducted for three U.S. Air Force personnel to compare the contribution of dermal exposure with the end-exhaled breath concentrations relative to inhalation exposure (Table 4). These three individuals were fuel-cell maintenance workers. The area under the end-exhaled breath concentration time curve (*AUC*_ex_) was calculated for dermal exposures using the following equation:


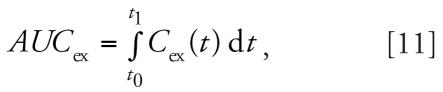


where *C*_ex_ is the concentration of naphthalene in the end-exhaled breath and *t*_1_ is the time at the end of the exposure. The values of *AUC*_ex_ for dermal exposures were 1.7, 41.7, and 521 μg × min/m^3^ for the 10th, 50th, and 90th percentiles, respectively. Dermal exposures were set to zero and the naphthalene air concentration was adjusted to obtain the same value of *AUC*_ex_. The predicted air concentrations (*INHAL1*_pred_) were 0.1, 0.7, and 11.7 μg/m^3^, respectively. These values are 1, 4, and 11% of the air concentrations of naphthalene for the individuals whose breath measurements represented the 10th, 50th, and 90th percentiles, respectively.

### Sensitivity analysis

**Figure 4:**
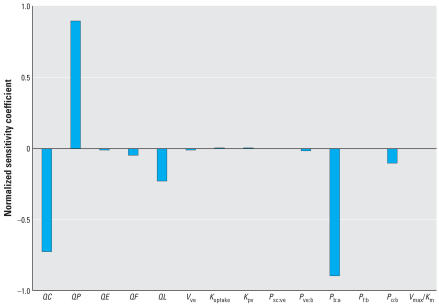
Normalized sensitivity coefficients for the end-exhaled breath concentrations. Parameters were adjusted at the 1% level.

Normalized sensitivity coefficients (mean) were calculated separately for exposure and physiologic parameters. The sensitivity analysis was conducted for a typical mixed exposure scenario, that is, the subject representing the 50th percentile in terms of end-exhaled breath measurements. Each parameter was changed 1% from its optimal value (Table 1) in the forward direction (Equation 8). The response variable in both sets of calculations was the concentration of naphthalene in the end-exhaled breath. For exposure variables, the end-exhaled breath concentration was most sensitive to the estimated air concentration of naphthalene during work (*NSC* = 1.0). End-exhaled breath concentrations were not sensitive to the variables *DERMDOSE* and *A*_exp_, as the dermal route accounts for only a small percentage of total exposure in these individuals. In the multidose route PBTK model, the end-exhaled breath concentration of naphthalene was most sensitive to cardiac output (*NSC* = −0.7), ventilation rate (*NSC* = 0.9), and the blood:air partition coefficient (*NSC* = −0.9) (Figure 4). The *NSC*s for other parameters were < |0.2|.

## Discussion

A PBTK model was developed to predict end-exhaled breath concentrations of naphthalene from dermal and inhalation exposure to JP-8. Our model consisted of five compartments representing the stratum corneum, viable epidermis, blood, fat, and other tissues, and contains fewer parameters than previously published physiologically based compartmental models of naphthalene (Quick and Shuler 1999; Willems et al. 2001). The fat was considered separate from the other tissues because the time constant for fat (8.6 hr) was larger than the time constant for other tissues (0.9 hr). However, the other tissue compartment was included in the model because the skin compartment consisted of the skin directly under the exposed area. The remaining skin was included in the other tissue compartment.

Adjustments were made to the fat:blood and other tissue:blood partition coefficients for the PBTK model predictions to fit the experimental and occupational exposure data. For many chemicals, the partition coefficients are not known. In such cases, quantitative structure–activity relationship (QSAR) models may be used to predict the necessary partition coefficients; however, the predictions are limited to chemicals with physicochemical properties that lie within the calibration data set (Beliveau et al. 2003). In our study we calibrated the values of *P*_f:b_ and *P*_o:b_, which were predicted by Willems et al. (2001) using QSAR models, against human exposure data. We estimated a *P*_f:b_ value of 25.6 for naphthalene, which is more plausible than 160 given that the *P*_f:b_ for benzene is 55 and 25 for decane. Using the vial equilibration technique of Gargas et al. (1989), we also measured a *P*_b:a_ value of 10.3 for naphthalene, which is more consistent with the *P*_b:a_ for other compounds than is the value of 571 reported by Willems et al. (2001). For example, the human *P*_b:a_ for benzene, cyclohexane, JP-10, and *p*-xylene were 8.19, 1.41, 52.5, and 44.7, respectively (Gargas et al. 1989).

The PBTK model was used to calculate the permeability coefficient (*K*_p_) for naphthalene in humans *in vivo*. Previously, the *K*_p_ had been calculated using Fick’s law of diffusion. A *K*_p_ value of 5.1 × 10^−4^ cm/hr was estimated for rat skin *in vitro* (McDougal et al. 2000). This *in vitro K*_p_ value was compared with a *K*_p_ value that was estimated by calculating the flux value for aromatic and aliphatic components of JP-8 in humans from the slope of the linear portion of the cumulative mass of chemical in blood per square centimeter versus time curve (Kim et al. 2006a). We calculated an apparent *K*_p_ of 5.3 × 10^−5^ cm/hr, which is approximately an order of magnitude lower than that for the rat *K*_p_. This *K*_p_ calculation was revised using a DTK model and Equation 9. A larger *K*_p_ value was estimated (1.8 × 10^−3^ cm/hr), which was more similar to the *K*_p_ estimated *in vitro* by McDougal et al. (2000). The limitation of using a data-based compartmental model is that the parameter values are not constrained by the actual anatomy and physiology of the human body and the biochemistry of naphthalene *in vivo*. We incorporated such constraints into our PBTK model and revised our calculation of *K*_p_ for naphthalene. We estimated a *K*_ps_ value of 6.8 × 10^−5^ cm/hr and a *K*_pv_ value of 3.0 × 10^−3^ cm/hr. The value of *K*_eff_, which is the overall permeability coefficient for chemicals crossing the skin (McCarley and Bunge 2001), is 6.6 × 10^−5^ cm/hr.

*K*_eff_ is approximately 7-fold smaller than the *K*_p_ reported by McDougal et al. (2000). A 7-fold difference was not unexpected because rat skin used in the McDougal et al. (2000) study is generally considered more permeable than human skin. Molecular diffusion is the dominant mechanism that governs the permeation of naphthalene across the skin. For diffusion, the flux (and *K*_p_) is inversely proportional to the thickness of the diffusion distance, as stated by Fick’s first law of diffusion. Therefore, doubling the thickness of the skin will result in halving the *K*_p_. McDougal et al. (2000) estimated *K*_p_ across rat skin of thickness 560 μm. The human skin thickness ranges from 500 μm to 4,000 μm; therefore, the human *K*_p_ value is expected to be between 6.4 × 10^−5^ cm/hr and 5.7 × 10^−4^ cm/hr. Our estimate of the effective permeability coefficient for naphthalene lies within this range of expected values.

The optimized PBTK model was used to predict end-exhaled breath measurements collected in the workplace for the U.S. Air Force personnel exposed to JP-8 by the inhalation route. The predicted concentration at the end of their work shift was the same as the measured values. Further comparisons of predicted versus measured values revealed considerable interindividual variability. Sources of heterogeneity in a population may include physical condition, level of activity, disease state, age, hormonal status, and interactions with other chemicals and drugs (Clewell and Andersen 1996). Further, we observed considerable variation in the values of *K*_ps_ and *K*_pv_, but the small sample size (10 subjects) limited the analysis of variability in our study. Further study of the heterogeneity of parameter values and the impact on the toxicokinetic profile of naphthalene in humans is needed.

We also used the optimized PBTK model to examine dermal and inhalation exposure to JP-8. Three U.S. Air Force personnel were selected who represented the 10th, 50th, and 90th percentiles based on their end-exhaled breath concentrations. The predicted concentrations of naphthalene were well above what was expected in end-exhaled breath. For example, for the personnel representing the 50th percentile, *INHAL1*_est_ overpredicted the end-exhaled breath concentration of naphthalene by 1,540% (i.e., 75.8 μg/m^3^ vs. 4.6 μg/ m^3^). The reason for overpredicting breath concentrations was that these workers wore personal protective equipment that included forced supply-air respirators while working in fuel tanks. Thus, the air concentration that was measured using the passive monitors was not the actual air concentration that the Air Force personnel were exposed to while working inside the fuel tanks.

The PBTK model was exercised to obtain a better estimate of the air concentration that corresponded to the breath measurements. The adjusted air concentration was used in our calculation of the relative contribution of dermal exposure to the end-exhaled breath concentration of naphthalene. We observed that the median contribution of dermal exposure to the end-exhaled breath concentration of naphthalene was relatively small (4%). However, in the U.S. Air Force personnel who represented the 90^th^ percentile, the relative contribution of dermal exposure to the end-exhaled breath concentration was 11%. The U.S. Air Force personnel examined in this study comprised fuel-cell maintenance workers. Thus, the use of dermal protective equipment can further decrease the end-exhaled breath concentration of naphthalene in the fuel-cell maintenance workers.

This PBTK model has reduced the uncertainty in modeling JP-8 exposures because fewer parameters were required to predict the time-course of naphthalene. However, our model has identified some data gaps. First, inhalation exposures should be measured over shorter time intervals. Sensitivity analysis demonstrated that end-exhaled breath levels of naphthalene were most sensitive to the air concentration of naphthalene during work. In our study, we used time-weighted average concentrations (over approximately 4 hr) that did not capture exposures to high levels of naphthalene from local sources. Therefore, shorter time-resolved data may be used to better explain the transient nature of inhalation exposures to JP-8. Second, occupational and environmental exposure studies of other components of JP-8 are needed to gain a more complete picture of JP-8 exposures. Currently, occupational exposure studies have focused on single chemical components of JP-8. The results of multichemical exposure assessment studies may be compared with results from single-chemical studies and add to our understanding of the absorption, distribution, metabolism, and elimination of complex chemical mixtures.

The modeling approach used in this study represents a useful technique for assessing the contribution of dermal and inhalation exposures to the systemic levels of chemicals. One of the primary applications of this work could be to improve the understanding of exposure processes by quantifying the relationship between external exposure measurements and biomarkers of internal dose. For example, a series of air and dermal exposure measurements may be collected from a sample of individuals from groups stratified by fixed factors such as location relative to the source of exposure. One could, for example, introduce an intervention (e.g., respirators) and use the PBTK model to quantify the efficacy of the intervention for reducing systemic levels of the toxicant. This approach would be useful for protecting the health of individuals. For example, if the concentration of the exposure biomarker (i.e., blood and/or breath concentration) is driven primarily by the dermal route in a given group, there would be little advantage in additional respiratory protection. This approach may be used in both occupational and environmental risk assessment applications. However, additional modeling and experimental studies are required before generalization of this model to confirm scenarios/dose metrics beyond the limitations of the current study.

In conclusion, we used the PBTK model to quantify the contribution of dermal exposures to the systemic levels of naphthalene. We estimated a permeability coefficient that was 7-fold lower than estimates for rat skin made *in vitro*. Our approach used a combination of exposure assessment, biological monitoring, and toxicokinetic modeling tools to integrate external exposure and biomarker data into a single description of the toxicokinetic behavior of naphthalene. The PBTK model incorporated exposures from both dermal and inhalation routes and required estimation of fewer parameters than previously published PBTK models of naphthalene. This PBTK model, which included two major exposure routes relevant to occupational and environmental exposure scenarios, may be used for integrating animal and human observational studies into an improved understanding of human health risks for JP-8. A wide range of permeability coefficient values was noted in the individuals in this research and further study of the sources of inter- and intraindividual variation in these processes appears necessary.

## Figures and Tables

**Table 1:** Naphthalene PBTK model parameters.

Parameter	Symbol	Unit	Value	Notes and references
Body weight	*BW*	kg	61	Kim et al. (2006a)
Height	*HT*	cm	174	Kim et al. (2006a)
Body mass index	*BMI*	kg/m^2^	20	*BMI* = *BW*/*HT*^2^
Organ volumes				
Blood^a^	*V*_b_	L	4.5	*BW* = (72.447/1000) × *BW*^1.007^
Stratum corneum	*V*_sc_	L	2 × 10^−5^	*VD* = *A*_exp_ × *Td*
Viable epidermis^b^	*V*_ve_	L	1.9 × 10^−3^	*VE* = *VEC* × *BW*−*VD*
Fat^c^	*V*_f_	L	5.5	*VF* = *BW* × (ln *BMI*−126.2)/100
Other tissue	*V*_o_	L	51.0	*VO* = *BW*−(*VB* + *VD* + *VE* + *VF* )
Pulmonary ventilation rate	*QP*	L/hr/*BW*^0.75^	15	Brown et al. (1997)
Cardiac output	*QC*	L/hr/*BW*^0.75^	15	Brown et al. (1997)
Regional blood flow				
To skin^d^	*QE*	L/hr	1.7 × 10^−2^	*QE* = *QEC* × (*A*_exp_/*SURFA*)
To fat	*QF*	L/hr	16.4	*QF* = *QFC* × *QC*
To other tissues	*QO*	L/hr	311.0	*QO* = *QC*−(*QE* + *QF*)
Metabolic clearance parameters				
Ratio of *V*_max_:*K_m_*	*V*_max_/*K_m_*	L/hr	698	Willems et al. (2001)
Blood flow to liver	*QL*	L/hr	75.3	*QL* = *QLC* × *QC*
Partition coefficients				
Blood:air	*P*_b:a_	—	10.3	Measured^e^
Stratum corneum:viable epidermis	*P*_sc:ve_	—	1.8	McCarley and Bunge (2001)
VE:blood	*P*_ve:b_	—	2.8	McCarley and Bunge (2001)
Fat:blood	*P*_f:b_	—	25.6	Estimated^f^
Other tissue:blood	*P*_o:b_	—	5.2	Estimated^f^
Skin permeation parameters				
Area of exposure	*A*_exp_	cm^2^	20	Dimensions of the tape strip
Thickness of the stratum corneum	*Td*	μm	10	McCarley and Bunge (2001)
Total body surface area^g^	*SURFA*	cm^2^	19,238	(*BW*^0.45^) × (*HT*^0.725^) × 71.84
Permeability coefficient for stratum corneum	*K*_ps_	cm/hr	6.8 × 10^−5^	Estimated^f^
Permeability coefficient for viable epidermis	*K*_pv_	cm/hr	3.0 × 10^−3^	Estimated^f^

a From Davies and Morris 1993.

b The volume of the viable epidermis is calculated as the volume of the exposed skin minus the volume of the stratum corneum under the exposed area. The fraction of body weight in skin (VEC) is from Brown et al. (1997).

c The fraction of body weight in fat = ln *BMI*−126.2 (Mills 2005).

d The fractions of cardiac output to skin (QEC) and to liver (QLC) were obtained from Brown et al. (1997).

e The blood:air partition coefficient was measured using the vial equilibration technique (Gargas et al. 1989).

f Model parameters were estimated by fitting the model to the data (Figure 2).

g Total body surface area (Haycock et al. 1978).

**Table 2:** Optimized values of the skin parameters *K*_uptake_, *K*_pv_, *P*_f:b_, and *P*_o:b_. *K*_ps_ were calculated using Equation 10. The parameters were optimized for each of the 10 study volunteers.

Volunteer	*K*_uptake_ × 10^−3^ (hr^−1^)	*K*_ps_ × 10^−5^ (cm/hr)	*K*_pv_ × 10^−3^ (cm/hr)	*P*_f:b_	*P*_o:b_
1	190.7	18.8	7.6	4.4	0.6
2	4.4	1.1	1.5	0.1	0.6
3	13.6	8.0	0.6	1.6	8.9
4	21.3	3.2	3.1	15.2	2.4
5	16.9	11.8	2.4	15.4	12.2
6	22.8	11.7	1.7	3.1	8.3
7	12.2	6.8	2.1	22.4	2.7
8	18.6	4.1	5.4	11.1	16.1
9	8.3	1.5	2.2	7.3	0.3
10	3.7	1.3	3.5	175.7	0.1
Mean ± SD	31.3 ± 56.4	6.8 ± 5.8	3.0 ± 2.1	25.6 ± 53.2	5.2 ± 5.8

**Table 3:** Input parameters and values for prediction of end-exhaled breath concentrations of naphthalene in the U.S. Air Force personnel who represented the 10^th^, 50^th^, and 90^th^ percentiles based on end-exhaled breath measurements.

	Percentile		
Variable	10th	50th	90th
Height (cm)	175	188	168
Body weight (kg)	81	109	73
*INHAL1*_est_ (μg/m^3^)	499	322	3,640
*INHAL2*_est_ (μg/m^3^)	2.0	2.0	2.0
*DERMDOSE* (μg/cm^2^)	3.9 × 10^−5^	5.5 × 10^−4^	9.2 × 10^−3^
Duration of exposure (min)	224	322	260

**Table 4:** Estimated contribution of dermal exposure to the end-exhaled breath concentrations of naphthalene relative to inhalation exposure.^a^

Percentile	Breath (μg/m^3^)	*AUC*_ex_ (μg × min/m^3^)	*INHAL1*_adj_ (μg/m^3^)	*INHAL1*_pred_ (μg/m^3^)	Ratio (%)
10th	1.7	1.7	7.4	0.1	1
50th	4.7	41.7	18.8	0.7	4
90th	29.4	521	103	11.7	11

a This analysis was based on three U.S. Air Force personnel whose end-exhaled breath concentrations represented the 10th, 50th, and 90th percentiles. The ratio of *INHAL1*_pred_ to *INHAL1*_adj_ is a measure of the relative percent contribution of dermal exposure to the end-exhaled breath concentration.
